# Demented patients and the quandaries of identity: setting the problem, advancing a proposal

**DOI:** 10.1007/s40656-021-00365-y

**Published:** 2021-02-15

**Authors:** Giovanni Boniolo

**Affiliations:** 1 Dipartimento di Neuroscienze e Riabilitazione, University of Ferrara, Ferrara, Italy; 2 Civitas Vitae Research Centre at Fondazione OIC onlus, Padova, Italy

**Keywords:** Identity, Empirical approach to identity, Whole phenotype, Dementia, Moral decisions

## Abstract

In the paper, after clarifying terms such as ‘identity’, ‘self’ and ‘personhood’, I propose an empirical account of identity based on the notion of “whole phenotype”. This move allows one to claim the persistence of the individuals before and after their being affected by dementia. Furthermore, I show how this account permits us to address significant questions related to demented individuals’ loss of the capacity of moral decisions.

## Introduction

In the refreshed *American Diagnostic and Statistical Manual* (DSM-5)^1^ there is a new term: “Major neurocognitive disorder”. It replaces an old entry which has terrified and continues terrifying individuals around the word: dementia.^2^ Independently of how we agree to name the pathology, it deals with decline of memory, attention, learning, language, perception, and social cognition. It means that this condition has a great impact on the individual’s everyday life and independence, and on the relatives’ and carers’ too. Furthermore, it also has a non-negligible socio-economic impact. According to the WHO, “in 2015, the total global societal cost of dementia was estimated to be US$ 818 billion, equivalent to 1.1% of global gross domestic product (GDP). The total cost as a proportion of GDP varied from 0.2% in low- and middle-income countries to 1.4% in high-income countries”. This is a factor of the enormous and ever-increasing number of individuals who struggle with it: “Worldwide, around 50 million people have dementia, with nearly 60% living in low- and middle-income countries. Every year, there are nearly 10 million new cases. The estimated proportion of the general population aged 60 and over with dementia at a given time is between 5 and 8%. The total number of people with dementia is projected to reach 82 million in 2030 and 152 million in 2050. Much of this increase is attributable to the rising numbers of people with dementia living in low- and middle-income countries”.^3^

Not only does dementia currently provide a medical, socio-economical and existential threat, but also it poses doubts about the demented patients’ persistence of identity. This essay is focused precisely on this issue and is intended to be a contribution to the vivid debate concerning the impression that dementia brings loss of the self, or loss of identity, or loss of personhood. This loss is alleged to occur since dementia negatively affects, step-by-step, the proper functioning of the main higher mental functions (memory and decision-making capacity). This decline, especially in the middle and late stages,^4^ could lead to the idea that the self, or the identity, or the personhood, has completely changed and is replaced by a different one: before the disease, an individual had a given self, a given identity, a given personhood; after the disease, this would be changed so much that we could speak in terms of a different self, a different identity or a different personhood. Further on, that decline could lead also to the idea that demented individuals would no longer have moral status, since they would no longer be persons.

What does it mean to speak about ‘self’, ‘identity’ and ‘personhood’? Before addressing the problem of whether a demented individual has a loss of self, identity, or personhood, and then provide a judgment about his or her moral status, we should have a clear idea of what we mean by those terms. Thus, in what follows, I first provide some clues to clarify the meaning of the terms. Next, without using vague or ambiguous term like “self” and “personhood,” I propose an empirical perspective on identity, based on the notion of ‘whole phenotype’ (I call it the *Whole Phenotype Account*, or WPA), which allows me to argue that the demented individuals maintain their identity, in particular their *whole phenotype identity*. To conclude, I advance some remarks on how it is possible to use the WPA to cope with the questions related to demented individuals’ loss of the capacity to make moral decisions.

## Setting the problem

Without purporting to analyze and assess all the numerous different definitions, criteria and tests^5^ adopted over centuries to clarify what self and personhood mean and what they have to do with the identity problem, I wish to survey the complex semantic bush in which the issue of dementia had been framed.

### Dementia: loss of what?

Some authors claim that dementia involves a *loss of the self*. What do they mean? In an excellent review, Caddell and Clare (2010)^6^ show how there is no agreement concerning what the self is and what the loss of the self involves. Moreover, they point out that there is debate “as to what extent the self persists or diminishes in people with dementia. Some researchers contend that the self remains intact throughout the course of dementia, while others insist that the self disintegrates ‘until nothing is left’. Many others fall in between these two extremes and suggest that the self is maintained to an extent, although compromised in some way”. To present the different positions, they distinguish *qualitative approaches* from *quantitative approaches*. The former “focus on verbal interactions involving people with dementia, but some concentrate on the non-verbal behavior exhibited by participants” as in the case of approaches concerning social construction, interaction, embodiment of selfhood, personal narratives and thematic analyses; while the latter use “experimental techniques and questionnaire measures” and are exemplified by approaches concerning autobiographical memory, role identities, self-recognition and self-knowledge. At the end of their analysis, they conclude that, given the variety of definitions, criteria and tests concerning ‘self’, there is no clear shared idea of what “loss of the self” should mean. This result about the vagueness of the term at stake does not seem a very encouraging starting point for a discussion concerning the demented individuals’ moral capacity or the persistence of their identity, if we wish to connect these issues with the self.

Other authors claim that dementia involves a *loss of personhood*, i.e. the status of being a person. Here the situation is much more complex. First of all, it is worth noting that many discussions underestimate the historical ambiguity of the notion of ‘person’ since its introduction into philosophical and psychological debates.^7^ In brief, ‘person’ (*persona*) entered the Roman law to indicate the recipient of those rules which do not pertain to things (*res*). From this point of view, ‘person’ is synonymous of ‘human being’ (*homo*) from his or her birth onward.^8^ That term was inserted in western philosophy through Christology when, probably for the first time, Quintus Septimius Tertullian borrowed ‘person’ from the legal language and introduced it into a theological and philosophical domain, defining Trinity as *tres personae, una substantia* (three persons, one substance).^9^ Within the Christian tradition, being a person has gradually and colloquially been understood as having a soul. Since those times, however, the philosophical discussion on person and personhood has been very rich and complex and many different proposals have been advanced. For example, there are those following Boëthius’s idea according to which person is "an individual substance of a rational nature" (*naturae rationalis individua substantia*). Another important tradition, especially for many Anglo-American philosophers, is the one begun with René Descartes, John Locke and David Hume according to which a person is any human being (and, according to some authors, also any non-human being) possessing higher mental functions (in particular, memory, consciousness and self-consciousness) allowing autonomous decisions, especially moral autonomous decisions. So, what is it to be a person? What is necessary and sufficient for a living being^10^ to count as a person, as opposed to a non-person? Being just born and being a member of the species *Homo sapiens*? Having a soul? Having certain higher mental functions? Which functions and in which degree?

On the one hand, we have the semantic vagueness of the notion of self and, on the other hand, we have the historical ambiguity of the notion of personhood. And what about the problem of*identity*? This issue involves two different but intertwined problems: the *who-problem*: “Who is a human being?”,^11^ that is, “Which are the features that make that human unique and different from others?”; and the *persistence-problem*: “What does it take for a human to persist from one time to another?”; that is, “Which are the features allowing us to affirm that that human is the same over time even if other properties have changed?”. This latter problem deals with diachronic identity, i.e. with numerical identity through time, or, in other words, with the fact that me as embryo, for example, and me as adult are the same human considered at two different times.^12^ Over the centuries, both the who-problem and the persistence-problem have been at the core of interesting analyses and vibrant discussions for their relevance to metaphysics, ontology, philosophical anthropology, and applied ethics. A number of solutions have been proposed for both problems. For example, if the question “Who am I?” is answered with “I am my higher mental functions”, as the supporters of the so-called *psychological approach* claim, then facing the persistence-problem means finding a criterion concerning a suitable type of psychological continuity (memory continuity, consciousness continuity, etc.). On the other hand, if the same question is answered with “I am my biological organism”, as the supporters of the *biological approach* claim, then dealing with the persistence-problem means searching for a criterion regarding the organism continuity. Both positions, however, have some weaknesses. The details of this very rich debate are beyond the scope of this article.^13^ It is, however, worth recalling that the psychological party has to face the objection that an individual should cease persisting or should not begin persisting if his or her memory or consciousness ceases to work, or never starts working, respectively. Thus, a fetus would not have begun persisting and a human in persistent vegetative state, or affected by a severe dementia, would have stopped persisting: a position that attracts considerable opposition on both deontological and consequentialist grounds. The biological approach seems to be too poor to be accepted as a criterion in case of human individuals. Usually, it is said that we—human individuals—are “more” than mere organisms.

Is it possible to find a third view that avoids the disadvantages of both the psychological and biological account of identity? I will show that the WPA allows exactly this possibility. Moreover, I will argue that the WPA also circumvents the quandaries of the definitory difficulties concerning ‘self’ and ‘personhood’.

### Dementia and personhood: the classical positions

We have seen that it is not so clear what the self is and that the history of the concept of personhood is too easily forgotten and too often restricted to something concerning some higher mental functions. Let us stay on these mental functions and try to understand whether we can make some sense of the everyday way of speaking in hospitals, in nursing homes, and even at home, when demented individuals are the object of discussion. In these situations, we easily hear relatives or carers say that “S/He is no longer the person s/he used to be”, as if there were two different individuals.^14^

The underlying problem concerns identity and it has been technically discussed by some philosophers who have, however, provided discordant solutions.^15^ Here, I recall two paradigmatic and classical positions. The first one was advanced by Dresser (1986) starting from a psychological account of identity mixed with a psychological characterization of personhood. According to her, a demented individual is totally different compared to the individual they were before the disease. Therefore, what they decided when they were healthy (for example, their advance directives) is no longer valid after the disease and we should take into consideration only the decisions that they take now (if they have the capability of making a decision!). That is, before there was a healthy individual with their choices and decisions; then there is a different, even if demented, individual with new choices and decisions and we should take these latter into consideration. Between the healthy individual and the demented individual there would not be psychological continuity, and this is relevant also concerning which decisions and which choices should be accepted by the relatives and the carers. By the way, it is worth noting that Dresser did not offer any discussion or identification of the moment in which there should be such a break in the psychological continuity. And this could be a problem for her account.

A different position is offered by Dworkin (1993), who argued that the healthy individual’s choices and decisions should take priority over the demented individual’s new choices and decisions. This position is based on the difference between “experiential interests” (i.e., avoiding pain, cooking or eating well, playing sports, watching movies, etc.) and “critical interests” (which depend on what we regard to be good, or worthwhile, or meaningful and having the significance of our entire life). Thus, since a demented individual could have only experiential interests, we should take into consideration only the critical interests they had when healthy. Again, there is the problem concerning the fact that there is no discussion nor identification of the moment in which the critical interests cannot be any longer pursued. Beyond this weakness, Dworkin has been much criticized, and not only by authors like Dresser (1995) who, nevertheless, share a common vision of the identity based on psychological continuity. The strongest criticism arrived from Jaworska (1999), who argued that the psychological continuity is not broken in many cases, especially in the early and middle stages of dementia (actually, Jaworska referred only to Alzheimer patients). Further on, even if it was broken, according to Jaworska, Dworkin’s distinction between experiential interests and critical interests, on the one hand, would be too strong and, on the other hand, we could consider forms of limited autonomy due to dementia.

A point is in order and it is a follow up on the Jaworska’s main argument against Dworkin. As noted, Jaworska emphasizes that, especially in early and middle stages of Alzheimer, the cognitive capacities of a patient are not completely lost, and the latter maintains a certain degree of memory and decision-making ability. Thus, especially at the beginning of the pathology, the psychological continuity is not broken, even if it is more and more fragile and feeble. Now we know something more on psychological continuity and severe dementia. Several studies have shown that there are “lucid episodes” also in individuals with severe dementia (see Normann et al. 2006; Mashour et al. 2019; Eldadah et al. 2019). This would mean, if we accepted an approach *à la* Dworkin, that we should consider a demented individual in a moment of lucidity as the same individual as they were when healthy (and therefore we should accept their choices and decisions), while in the non-lucid moments as a different individual (and therefore we should not accept their choices and decisions). Interesting position, but rather bizarre and difficult to justify.^16^

## Going for a different path

After sketching the state of the discussion, it is time to find a way out that allows us (i) to avoid the definitory vagueness of the notion of self and the historical ambiguity of the notion of personhood; (ii) to continue speaking of identity persistence also for demented individuals; (iii) to pave the way for potential solutions of the question concerning demented individuals’ moral status. These three outcomes will be achieved by introducing the WPA of identity, based on the notion of ‘the whole phenotype’, which, moreover, overcomes the problems both of the biological (even if WPA is grounded in biology) and of the psychological account.

### The WPA of identity

Boniolo and Testa (2012) proposed an empirical account of identity based on the notion of ‘whole phenotype’. Borrowing from the evo-devo (evolutionary/developmental) line of thinking, by ‘whole phenotype’ I mean the set of all those strongly intertwined phenotypic modules (the metabolic phenotype, the immunological phenotype, the nervous phenotype, the somatic phenotype, the behavioral phenotype, etc.)^17^ that render an individual what they are at a given time of their development. Here the notion of ‘phenotype’ deserves to be clarified, since, over the decades, many different definitions have been proposed. I use it in the contemporary biological meaning, that is, as something referring both to gene expression and regulation as a consequence of (internal and external) signals (also due to the environment in which one lives and to its life style), and to DNA methylation and selected histone post-translational modifications, that are persistent over many cellular generations independently of the underlying DNA sequence. That is, the phenotype we have at a certain time of our life is the product of all the genetic and epigenetic processes happened since the moment of fecundation. Moreover, always following an evo-devo perspective, it should be emphasized that ‘development’ regards all the processes and changes occurring in an individual over his or her lifespan and then affecting different phenotypic modules (and, as a result, his or her whole phenotype). In this way, ‘development’ is a process occurring all through life and, thus, a continuous move from a past whole phenotype (for example, the embryo whole phenotype) to the current whole phenotype (for example, the newborn whole phenotype, or the adult whole phenotype).^18^

Now, we could claim that a human individual in any instant of their life, is nothing but the result of all the genetic and epigenetic processes that, in the course of time, have causally shaped all of their intertwined phenotypic modules (be they metabolic, somatic, immunological, nervous, behavioral, etc.), that is, *an individual, in any instant of their life, is nothing but their whole phenotype*. This means, coming to the persistence problem, that *an individual’s identity through time is given by the continuity of their whole phenotype.*

It is not necessary now to go through all the technical details and the justification of the proposal (for more detail, see Boniolo and Testa 2012). Nevertheless, for the sake of current discourse, among the many phenotypic modules composing the whole phenotype, it is worth dwelling upon the central nervous phenotype, which allows lower and higher mental functions.

To be scientifically sound, we should affirm that an individual’s higher mental functions are what they are because of a particular instantiation of their central nervous phenotype, which is, in turn, the result of all the genetic and epigenetic processes occurred until that moment of their development. That is, no individual can have the higher mental functions they have without the suitable central nervous phenotype, as, for example, the extreme cases of the anencephalic infants, patients in persistent vegetative state, or demented individuals show.

To sum up, the higher mental functions possessed by a given individual are the result of their central nervous phenotype and this is, in turn, the result of their genetic and epigenetic history. Needless to say, by taking into consideration that we are moving along a purely empirical approach, we are implicitly affirming that possessing particular higher mental functions without the suitable nervous phenotype is impossible.^19^

Accepting this scientific perspective means overcoming the mentioned difficulties affecting both the biological account and the psychological account of identity. Concerning the former, according the WPA we are not only our organism, but also (i) the allowed and correlated central nervous phenotype, and therefore the allowed and correlated higher mental functions (memory, consciousness and self-consciousness), and (ii) the allowed and correlated behavioral phenotype. Concerning the latter, the WPA allows us to positively cope with the identity of embryos, fetuses, patients in persistent vegetative states and demented individuals, because we are not only our mind, in particular our higher mental functions, but also all the other phenotypic modules composing our whole phenotype. It is to note that the WPA is surely grounded in biology: it speaks in terms of phenotypes due to genetic and epigenetics processes. Nevertheless, since it considers the nervous phenotype and the correlated higher mental functions, it goes beyond a purely organismic account and regards also the aspects characterizing the psychological account. In different terms, WPA should be thought of as a bridge between the organismic account and the psychological account; in particular it is an account that takes into consideration the relevant aspects of both these accounts, without having their weaknesses.

Concerning our central question, that is, the link between identity and dementia, if we accept the WPA we can continue speaking about the persistence of demented individuals’ identity. They can have a brain pathology, and this could be called ‘dementia’ or ‘major neurocognitive disorder’, but this does not imply that they have lost their identity over time. They could have lost some higher mental functions, due to some brain damages, but this does not affect their identity over time, since this is guaranteed by the continuity of their whole phenotype, even if a phenotypic module (in particular, the central nervous phenotype) is changed in a pathological manner. There is no reason—and this is my main point—to privilege a particular phenotypic module over the others. No one, I suppose, would claim that we are not the same individual if we had a severe cardiac disease, or a severe immunological disease. The cardiac phenotypic module or the immunological phenotypic module would be, respectively, changed in a pathological way; some of the original functions would be lost; but this would not imply the loss of the identity as a whole. The same considerations should be applied to the nervous phenotype: it can be damaged by a disease, but this does not mean that we have lost our identity over time.

At this point we have the possibility to speak about individuals with dementia without speaking about their loss of identity, unless we prefer to embrace a particular theory of identity based only on the higher mental functions. But, as seen, there is no necessity to adhere to such a position. There is no need to claim that if an individual has lost the memory of who they were, they are no longer the same. Actually, they are the same from the WPA point of view, even if they do not know who they were. Memory is one of the functions of a non-pathological central nervous phenotype, which, in turn, is one of the many phenotypic modules composing the whole phenotype. An analogous discourse could be constructed for behavior. Individuals with some level of behavioral impairment could have totally different conducts from those ones they had before the pathology. But, again, this does not mean they are different individuals. The behavioral phenotype has changed (and of course the social relationships, as a consequence), but this is one of the many phenotypic modules composing the whole phenotype. Both in the former and in the latter case we have moved continuously from a (pre-dementia) whole phenotype to a (early, middle or severe dementia) whole phenotype.

We could speak in terms of identity of single phenotypic modules: we could say that there is the question of the identity of central nervous system, as there could be the question of the identity of the immunologic system, of the metabolic system, etc. Nevertheless, none of them implies the identity of the individual as a whole, since their identity has to be given in terms of their whole phenotype and there is no reason at all for choosing a particular phenotypic module over the others. We speak in terms of identity of higher mental functions, but we should recall that they are an expression of the brain we have, that is, of the central nervous system we have. The higher mental functions are not the individual as a whole.

Up to this point, I have introduced the notion of whole phenotype and argued for a theory of identity based on its continuity. Accepting this account means accepting that a demented individual has not had any break in their identity, even if they have lost their cognitive capacities, in particular the decision-making capacities. It means accepting that they have a peculiar disease and not that they are something else from what they were before.^20^

## Ethical considerations

In literature, we can find a conspicuous debate concerning how and why the problem of the identity of demented individuals should be connected with moral issues. In particular two topics are of interest here: (i) moral responsibility (“What allows us to claim that a demented individual is morally responsible for his or her actions?”); (ii) decision maintenance (“What allows us to claim that a demented individual’s advance directives have to be fulfilled?”).^21^ Most answers rely on the notions of self or personhood, as, for example, Dresser and Dworkin propose. Yet, could the WPA offer a different view?

### Moral responsibility

Moral responsibility regards both what we are (or are not) doing now (i.e. responsibility for present events) and what we did (or did not do) (i.e. responsibility for past events). This is not the place to propose or discuss any theory of responsibility and, therefore, which events (actions, omissions, consequences of actions, consequences of omissions, etc.) we should be held responsible for.^22^ For our present purposes, it is enough to note that, in order to be responsible for something, an individual has to be capable of moral judgments on what the right and wrong actions/omissions are and, possibly, of behaving consequently. Trivially enough, in order to have this capacity an individual has to have the suitable central nervous phenotype.^23^ This is the reason why an anencephalic infant, a patient in a persistent vegetative state or with brain pathologies like severe dementia cannot be morally responsible for their actions/omissions.

Having or not having a suitable central nervous system to allow moral responsibility is not something affecting the persistence of identity, if we accept the WPA. In the case of dementia, there is a more or less continuous decline of the cognitive capacities, in particular of the capacities of moral judgment, which is, in turn, the consequence of the continuous worsening of brain damages. Nevertheless, as said, this affects one of the many phenotypic modules composing the whole phenotype, but it does not affect identity. That demented individual incapable of moral judgments is the same individual who existed before the severe dementia, even if now they have a disease which has destroyed this capacity. *They have a neuro-physiological problem, not an ontological problem.* They have a physical disease resulting in considering them without moral responsibility; but they do not have an “ontological disease” resulting in considering them different from what they were before the physical disease!

### Decision maintenance and advance directives

Advance directives concern the possibility that an individual capable of decision making and free of any constrain could convey their wishes to family, friends, health care professionals, or legal representatives concerning situations in which, due to pathologies or accidents, they had no longer the proper capacities of decision making.

Let us consider two problems: (i) “Has an individual the right to change their mind and to withdraw their advance directives?”; (ii) “Has an individual the right that their advance directives be maintained?”. As noted, these were the problems that, for example, Dresser and Dworkin linked to the question of identity.^24^ But is really necessary such a link?

Let us suppose that an individual has signed the advance directives when they had the proper central nervous phenotype to allow the proper capacity of being a decision maker. Let us suppose that, at certain moment of their life, dementia begins its terrible course. Are they the same individual in the course of the dementia degeneration? From the point of view of the WPA, they are the same individual, even if they, step by step, lose their capacity to be a decision maker. In particular, they are affected by a disease characterized by an irreversible decline of certain brain functions due to the irreversible damaging of certain parts of the brain; nevertheless, they are the same individual from the point of view of the WPA, differently from what defended by Dresser and Dworkin.

Notwithstanding their common idea that there are two different individuals, Dresser and Dworkin diverge on the conclusions. According to Dresser what a healthy individual decided when they were healthy is no longer valid after they have lost the capability of decision-making; according to Dworkin, instead, the decisions and choices before the cognitive decline should take priority over the demented individual’s new choices and decisions. In my view, here we have two questions which should be taken well separate: one concerning demented individuals’ identity and one concerning the advanced directives. The former, as seen, has a solution from the point of view of WPA: we have only one individual. The latter, instead, should be connected with the capacity of being an autonomous decision-maker. Concerning this issue, I would be inclined to claim that, as long as they have sufficient capacities to be a decision maker, they have both the right to withdraw the advanced directives and to maintain them; but when they have reached a stage of dementia characterized by the total loss of the capacity of being a decision maker,^25^ the advanced directive should be maintained. This maintenance should not be based on the fact that *they have lost the right* to withdraw (or on something concerning their identity), but on the fact that *they have lost the possibility* to withdraw with the necessary complete cognitive awareness. Nevertheless, they should maintain the right that their wishes expressed before the disease be implemented. Moreover their relatives and carers should have the duty to satisfy this implementation.

## Conclusions

Dementia, or major neurocognitive disorder, is a severe disease with a great impact on people’s life both at individual and a collective level. Furthermore, with the increase of the life span, an increasing number of people is affected by this terminal pathology: there is no cure for dementia, indeed.^26^

Our attention to dementia should also be conceptual, since this attitude could help in understanding what it is and how to deal with it, at least from the point of view of the care (and not only of the cures) that a demented individual needs.

The proposed conceptual analysis has resulted, on the basis of the WPA, in showing that there are no strong reasons to claim that a demented individual is a different individual compared to whom they were before the disease. Claiming that they are different means starting from very partial accounts of identity where, many times, vague concepts (as self), or ambiguous concept (as personhood) are introduced. We do not need this kind of accounts when we are in the unlucky position of being forced to consider the dementia of our relatives. We need an open mind to understand that the demented individual in front of us is not a different individual from what they were before: they are the same, as the WPA allows to argue. Unfortunately, they have a terrible pathology and we have to help them and take care of them as far we can and until we can. We have to respect them and their choices and decisions as long as they are capable of choosing and deciding. Then, when this capacity has vanished, we have to continue respecting not only them, but also the choices and decisions they made.

## Footnotes

1 See American Psychiatric Association (2013).

2 In the following, I will use the locution ‘individual(s)’ instead of ‘person’, ‘human being’, etc. Moreover, where necessary, I will use the *singular they* (and its derivative forms) as a gender-neutral singular pronoun.

3 https://www.who.int/news-room/fact-sheets/detail/dementia; https://www.who.int/mental_health/neurology/dementia/dementia_thematicbrief_epidemiology.pdf (Access October 2020).

4 The middle stage involves “becoming forgetful of recent events and people's names; becoming lost at home; having increasing difficulty with communication; needing help with personal care; experiencing behaviour changes, including wandering and repeated questioning”. Instead, the late stage means “becoming unaware of the time and place; having difficulty recognizing relatives and friends; having an increasing need for assisted self-care; having difficulty walking; experiencing behavior changes that may escalate and include aggression”. These two definitions are taken from https://www.who.int/news-room/fact-sheets/detail/dementia (Access October 2020).

5 As is well-known, definitions, criteria and tests are different things. In short, a definition is the way of giving the meaning to a locution; a criterion is an abstract procedure by means of which we know to what the defined locution applies; a test is an empirical procedure by means of which we assess if the criterion at issue is satisfied in the given empirical context. About these three concepts, see Boniolo (2007).

6 See also, Eustache et al. (2013) and Hughes et al. (2006).

7 For an analysis of the misunderstanding arising from the conflation of different meanings, see Boniolo et al. (2007).

8 Thus, the *ius personarum* concerns all the human beings (*omnes homines*) independently of their being free or slave. Nevertheless, according to the Romans, even if all human beings since their birth are persons, among the status *personarum* (i.e. the status of being a person), the *pater familias* (the family’s father) is hierarchically superior to all the other family’s members.

9 In that way, Tertullian’s intention was to render in Latin the meaning of the Greek term *hypostasis*.

10 But also for a non-living being according to some scholars, see Chisholm (1976) and Baker (2000).

11 The problem of identity does not concern only humans, but let us remain on these ones.

12 Therefore, the persistence-problem has nothing to do with 1) the *identification-problem* (or *evidence-problem*): this pertains to forensic sciences, since it concerns the features by means of which we can identify that particular human; 2) *qualitative identity*: this regards the possibility of possessing all the same features over time, which cannot occur with humans (and not only with humans) since they change over time.

13 This debate is extremely complex and any attempt of summarizing it in the space of a section is impossible. Both the psychological and the biological approaches are very fragmented in an almost endless series of variations. However, in order to have an idea, see, for example, Noonan (1989), Olson (1997, 2003), Parfit (1984), Shoemacher and Swinburne (1984), Strawson (1959), Unger (1990), Wiggins (2001), Blatti (2007), and Curtis and Noonan (2015). For an introduction, see Noonan and Curtis (2018), Shoemaker (2019) and Olson (2019).

14 Concerning what the people think their identities are constituted of, see Strohminger and Nichols (2014, 2015).

15 Reviews can be found in the essays contained in Hughes et al. (2006) and in Chiong (2013).

16 There is a second, more tangential, point. It concerns the fact that the philosophical discussion on identity and psychological continuity has been very often nourished with fictional examples (philosophical *Gedankenexperimenten*), which have complicated a lot the matter from an abstract point of view, but which are not so useful to discuss real cases where demented individuals really exist. For example, Parfit (1984) argues that numeric identity is not relevant while it is the psychological identity since there could be the (fictional) possibility that an individual could be in psychological continuity, in a possible future, with two individuals due to a “replicator”, or due to the fact that each one of these new two individuals has half of preceding person’s brain. In this case we would have not numerical identity but only psychological identity, differentiating in this way the two kinds of identity. Unfortunately (or fortunately) we should not meet in reality situation like that, and I would prefer to speak in term of real demented individuals more than in terms of “replicated” individuals. Moreover, it should be recalled that “psychological continuity” is a rather vague locution, if we do not specify which psychological continuity should continue (memory, consciousness, self-consciousness, others?). Moreover, different philosophers have treated differently these phycological features (see Taylor 1976; Bratman 2002). By the way, we should be aware that not all the psychological features have the same decline in the same time (see, for example, Byrne 1996; Brown 2017).

17 Concerning the phenotypic modules, any of them could be considered independently by means of a heuristic abstraction. For example, one could study only the metabolic phenotype, the immunological phenotype, the nervous phenotype, the behavioral phenotype, etc. Actually, none of them is really independent of the others, since all of them are strongly correlated in any stage of the development.

18 On the notion of development, see West-Eberhard (2003).

19 See, Changeux and Konishi (1987), Schlaggar and O’Leary (1991), Greenough and Black (1992), Nelson (1995, 2000), Singer (1995), Chan and Jan (1999), Greer and Greenber (2008), and Borrelli et al. (2008).

20 At this point, someone, when dealing with demented patients, could not be so much interested in ontological accounts of identity, as those discussed above, but in sociological, or existential, or narrative accounts. Nevertheless, I am not so sure that they add something completely new. Concerning the *existential approaches* to identity, one might see, for example, the work by Ricoeur (1990) and how Radden and Fordyce (2006) use it. Concerning the *narrative approaches* to identity, one might read Somers (1994), McAdams (2017, 2018) and De Grazia (2005). However, to make a more extensive example, let us dwell upon the *socio-relational account* of identity proposed by Baylis (2012), which is strongly inserted in a feminist framework. According to her “my identity is neither in my body (viz., the somatic or biological account of personal identity) nor in my brain (viz., the psychological account of personal identity) but, rather, in the negotiated spaces between my biology and psychology and that of others. These others include those who are a part of my familial, social, cultural, and political clusters of meaning and belonging, those who know me from a distance, and still others, near strangers, who do not know me”. It is certainly true that, from a sociological point of view, who I am strongly depends on my relationships with the “others”. But my individuality lies also in my body and in my psychology, that is, in my whole phenotype, to adopt WPA. I could both have an ontological identity and a socio-relational identity. There are no contradictions or incompatibilities between the two. Independently of the question concerning identity, however, the socio-relational accounts strongly emphasize a non-negligible issue: the net of social relationships which the individual is always inserted in. This relational net (or one of its subnets) could be particularly relevant in the early and middle stage of dementia (and even in the lucid episodes of a severe dementia). It is the net of relationships, in particular the subnet realized by the carers and the relatives, which could help in covering the memory gaps, or the other higher mental functions gaps. And this coverage could last until the demented individual has reached a stage where it is no longer possible to act to provide a support to their memory or to their higher mental functions.

21 Concerning the general question regarding the relationships between identity and ethics, see Parfit (1971, 1972), McMahan (2002), De Grazia (2003, 2005), Shoemaker (2007, 2010a, b), and Boniolo (2013).

22 For example, some authors defend the position that responsibility regards also what we should (or should not) do now and in the future. I must skip this problem now. Concerning actions and omissions, see Boniolo and De Anna (2006).

23 On this, see, for example, Rottschaefer (1998), Greene et al. (2004), and Greene (2012).

24 Needless to say, there is a vast literature on this topic, see for example, the seminal Buchanan (1988) and De Grazia (1999).

25 Here we have a serious problem: who has the right to decide that this point has been reached?

26 It was S.L. Mitchell who definitively showed that dementia is a terminal disease and that this fact is underrecognized also at clinical level; see Mitchell et al. (2009) and Mitchell (2015).
